# Drench Application of Systemic Insecticides Disrupts Probing Behavior of *Diaphorina citri* (Hemiptera: Liviidae) and Inoculation of *Candidatus* Liberibacter asiaticus

**DOI:** 10.3390/insects11050314

**Published:** 2020-05-16

**Authors:** Michele Carmo-Sousa, Rafael Brandão Garcia, Nelson Arno Wulff, Alberto Fereres, Marcelo Pedreira Miranda

**Affiliations:** 1 Fund for Citrus Protection, FUNDECITRUS, Araraquara, São Paulo 14807040, Brazil; m.sousatimossi@gmail.com (M.C.-S.); rafael.garcia@fundecitrus.com.br (R.B.G.); nelson.wulff@fundecitrus.com.br (N.A.W.); 2 Spanish National Research Council, CSIC, 28006 Madrid, Spain; a.fereres@csic.es

**Keywords:** Asian citrus psyllid, huanglongbing, chemical control, electrical penetration graph

## Abstract

*Candidatus* Liberibacter asiaticus (*C*Las) is a phloem-limited bacterium that is associated with the Huanglongbing (HLB) disease of citrus and transmitted by the psyllid, *Diaphorina citri*. There are no curative methods to control HLB and the prevention of new infections is essential for HLB management. Therefore, the objective of our study was to determine the effects of systemic insecticides, such as the neonicotinoids imidacloprid, thiamethoxam, and a mixture of thiamethoxam and chlorantraniliprole (diamide) on the probing behavior of *C*Las-infected *D. citri* and their effect on *C*Las transmission. The electrical penetration graph (EPG-DC) technique was used to monitor the stylet penetration activities of *C*Las-infected *D. citri* on sweet orange [*Citrus sinensis* (L.) Osbeck] ‘Valencia’ treated with systemic insecticides. Systemic insecticides disrupted the probing behavior of *C*Las-infected *D. citri*, in a way that affected *C*Las transmission efficiency, particularly by negatively affecting the stylet activities related to the phloem phase. All insecticides reduced (by 57–73%) the proportion of psyllids that exhibited sustainable phloem ingestion (waveform E2 > 10 min), with significant differences observed on plants treated with thiamethoxam and thiamethoxam + chlorantraniliprole. The transmission rate of *C*Las with high inoculum pressure (five *C*Las-infected *D. citri* per plant and a seven-day inoculation access period) to untreated control plants was 93%. In contrast, *C*Las transmission was reduced to 38.8% when test plants were protected by systemic insecticides. Our results indicated that all insecticides tested presented a potential to reduce *C*Las inoculation by an average of 59%; therefore, these insecticides can be used to reduce the spread of HLB.

## 1. Introduction

Citrus is one of the most important fruit crops worldwide; the citrus diseases caused by the pathogens transmitted by arthropod vectors are mainly responsible for the increasing citrus production costs. The Huanglongbing (HLB), also known as citrus greening disease, is the most devastating citrus disease threatening citrus production worldwide; this disease is associated with the *phloem-limited* bacteria, *Candidatus* Liberibacter spp. such as *Ca.* L. asiaticus (*C*Las), *Ca.* L. africanus (*C*Laf), and *Ca.* L. americanus (*C*Lam) [1]. HLB was first reported in the Americas in the State of Sao Paulo, Brazil, in 2004 [2,3] and later in the State of Florida, USA, in 2005 [4]. The disease has since then moved into the neighboring states of Brazil, Argentina, and Paraguay [5]. In Sao Paulo and West of Minas Gerais State, HLB has then reached epidemic proportions, and currently affects 19% of citrus trees [6]. In Brazil, HLB is reported to be associated with both *C*Lam and *C*Las; however, because *C*Las has higher titers than that of *C*Lam in the citrus trees, it is gradually overtaking *C*Lam [7,8]. *C*Las was detected in over 99% of HLB-infected trees in Sao Paulo State in 2013 [5]. Both these Liberibacters are transmitted by the Asian citrus psyllid, *Diaphorina citri* Kuwayama (Hemiptera: Liviidae) [9,10] during phloem-feeding on citrus trees.

Studies have demonstrated that adult *D. citri* that acquired *C*Las as nymphs are more efficient in inoculating the pathogen as compared to those that acquired it as adults [11,12]. After *C*Las acquisition, the bacterium was found in various organs and tissues of the insect, such as the midgut, hemocoele, ovaries, and salivary glands [13,14]. In general, the *Candidatus* Liberibacter spp. shares a specific and intimate relationship with their psyllid vectors, and its transmission characteristics indicate a persistent propagative pathogen–vector relationship [13,15], which is acquired and inoculated when psyllids ingest and salivate in the phloem tissues. In addition, factors such as temperature and vegetative stage of the leaves can improve *C*Las transmission; the presence of citrus flush [16] and temperatures ranging from 25–27 °C (S.A. Lopes, unpublished data) are highly favored by *D. citri* for *C*Las inoculation.

In the absence of any curative treatment for HLB, the disease is managed by planting healthy nursery trees, eliminating symptomatic plants for reducing the inoculum sources, and chemically controlling *D. citri* [17,18].

The probing behavior of hemipterans cannot be observed directly, however, it can be monitored in real time using the electrical penetration graph (EPG) technique [19,20]. The EPG technique is popular among researchers studying plant–hemipteran interactions and the transmission of plant pathogens by its insect vectors. The feeding behavior of insects such as aphids, whiteflies, leafhoppers, sharpshooters, and stink bugs have been studied by EPG [21,22,23,24,25]. This technique has been used to study the feeding behavior of psyllids, such as the European pear psylla *Cacopsylla pyri* (Foster) [26], the tomato and potato psyllid *Bactericera cockerelli* (Sulc) [27,28,29,30], the carrot psyllid *Bactericera trigonica* Hodkinson [31] and *D. citri* [32].

The probing behavior of *D. citri* is described by using five characteristic EPG waveforms (C, D, E1, E2 and G) and by dividing the phloem phase into three phases: waveform D (first phloem contact), waveform E1 (phloem salivation), and waveform E2 (phloem ingestion) [32]. The acquisition most likely occurs during phloem ingestion, and the inoculation during phloem salivation, as observed in previous studies with *C*Las and *D. citri* [32,33,34]; a likely association between the inoculation of *Ca.* L. solanacearum by *B. cockerelli* and salivation in the phloem sieve elements (waveform E1) was also reported by Sandanayaka et al. [35] and Antolinez et al. [31].

Systemic insecticides can promote changes in the feeding behavior of sap-sucking insects; some researchers reported that insecticides significantly disrupt the probing behavior of psyllids in plants treated with cyantraniliprole and abamectin [36], imidacloprid [37,38,39], and thiamethoxam [39]. However, all these studies were performed with psyllids free of *Candidatus* Liberibacter spp.

Therefore, in this study, we tested the effect of drench application of imidacloprid, thiamethoxam, and thiamethoxam + chlorantraniliprole on the probing behavior of *C*Las-infected *D. citri* on citrus nursery trees by using the EPG technique; we also measured the impact of systemic insecticides in *C*Las transmission after the psyllids fed on the insecticide-treated citrus nursery trees. The results of this study should provide a better understanding of the effects of systemic insecticides (neonicotinoids and diamide) on the probing behavior of *C*Las-infected *D. citri* and help refine the vector control strategies.

## 2. Materials and Methods

### 2.1. Plant Material and D. citri CLas-Infection

Sweet orange nursery trees, *Citrus sinensis* (L.) Osbeck ‘Valencia’ (70–80 cm tall and one year old), grafted on Swingle citrumelo, *Citrus paradise* Macf. × *Poncirus trifoliata* L. (Raf.), rootstock were kept in plastic bags (4 L) with Pinus substrate (MultplantCitrus®; Holambra, SP, Brazil) and used for further experiments. 

*Diaphorina citri C*Las-free colony was maintained on healthy orange jasmine plants, *Murraya paniculata* (L.) Jack, in insect cages under laboratory conditions (temperature, 26 ± 2 °C; relative humidity, 70%; and a photoperiod of 14/10 h of light/dark, respectively) at Fundecitrus, Sao Paulo, Brazil. The colony was confirmed to be *C*Las-free through multiple sampling tests by quantitative Polymerase Chain Reaction (qPCR) [7]. 

*C*Las-infected *D. citri* adults were obtained by keeping the *C*Las-free psyllids and *C*Las-infected (PCR-positive) sweet orange nursery trees confined together for the psyllids’ entire life-cycle (eggs, nymphs, and adults) in a room with controlled conditions (26 ± 2 °C, 70% relative humidity, and a 14/10 h light/dark photoperiod, respectively); this was performed before the start of the feeding behavior and transmission experiments. The colony was confirmed to be *C*Las-infected by qPCR through sampling tests [7]. All the psyllids used in the study had 10–15 days post-emergence. 

### 2.2. Insecticide Application

The sweet orange nursery trees from the same batch were pruned to promote shoot growth and immediately treated by drenching with the following insecticides: Imidacloprid (Provado® 200 SC; Bayer, Belford Roxo, RJ, Brazil) at 0.31 g active ingredient (AI) per plant, thiamethoxam (Actara® 250 WG; Syngenta, Paulínia, SP, Brazil) at 0.25 g AI per plant, and thiamethoxam + chlorantraniliprole (Durivo® 300 SC, Syngenta, Paulínia, SP, Brazil) at 0.2 + 0.1 g AI per plant. All the insecticides were diluted in 50 mL of water per plant and applied to the substrates. This is the same approach used by citrus growers before planting. Post-treatment, the trees were kept in a greenhouse for 20 days until the start of the experiments; untreated plants (control) were grown and pruned under the same conditions as that of the insecticide-treated plants.

### 2.3. Electrical Penetration Graph (EPG) Recordings

The probing behavior of *C*Las-infected *D. citri* was recorded using an EPG-DC device (Model Giga-8; EPG Systems, Wageningen, The Netherlands). The recordings were performed with 100X gain, and the signal was converted from analog-to-digital (A–D) using a DI 710 card (Dataq Instruments, Akron, OH, USA). The EPG data was acquired using a Duo core® desktop computer, and it was analyzed using Stylet+ software (EPG Systems).

*C*Las-infected *D. citri* females were anesthetized with CO_2_ for three seconds and immobilized individually using a vacuum-operated pump connected to a plate similar to that described by Bonani et al. [32]. Then, a gold wire (length, 2–3 cm; diameter, 18 µm) (Coining, Montvale, NY, USA) was placed on the insect pronotum with a small drop of silver glue (16034 Pelco Colloidal Silver, Ted Pella Inc., Redding, CA, USA). *D. citri* individuals, attached to the gold wire, were placed on the abaxial surface of the leaf (vegetative stage, V3) and monitored for 8 hours. The EPG recordings were performed in a climate-controlled room (25 ± 2 °C) with artificial light provided by six fluorescent lights (240 W) and were started immediately after the psyllids were placed on the leaf. A minimum of 40 recordings was performed for each treatment. Two plants from each treatment were randomly located in a Faraday cage per EPG recording. After each recording, the sweet orange nursery trees and *C*Las-infected psyllids were replaced and randomly arranged in the cage.

The EPG data was analyzed according to the waveforms described by Bonani et al. [32]: non-probing (np) behavior; intercellular apoplastic stylet pathway and salivary sheath secretion (C); first phloem contact (D); salivation into phloem (E1); phloem sap ingestion (E2); and xylem sap ingestion (G).

The output of EPG recordings given by Sarria et al. [40] (EPG-Excel Data Workbook) for each given *D. citri* replicate were used for calculating the treatment mean for each EPG sequential and non-sequential variables. The selected EPG variables (Mean ± SE) were calculated and compared among treatments as described by Van Helden and Tjallingii [41], Backus et al. [42] and Bonani et al. [32]. The selected variables are as follows: proportion of individuals which produced a specific waveform type (PPW); the number of waveform events per insect (NWEI), which is the sum of the number of events of a particular waveform divided by the total number of insects under each treatment; the total waveform duration (min) per insect (WDI), which is the sum of durations of a particular waveform divided by the total number of insects under each treatment; and the waveform duration (min) per event (WDE), which is the sum of the duration of events for a particular waveform divided by the total number of events of that particular waveform under each treatment.

After recording the EPG data, psyllids were stored at −80 °C until qPCR analysis for the presence of *C*Las. The nursery citrus trees were kept inside an insect-proof greenhouse, and the leaf samples were taken at six and 12 months after inoculation for qPCR detection of *C*Las.

### 2.4. Effects of Insecticides on CLas Transmission by D. citri

The experiment was conducted to determine the effectiveness of systemic insecticides in preventing and/or reducing the *C*Las transmission by increasing the inoculum pressure.

Twenty days before the start of the experiment, the sweet orange nursery trees were pruned and treated with the systemic insecticides as described before. Batches of five *C*Las-infected adults *D. citri* (10–15 days old) of mixed gender, were confined in a young shoot (vegetative stage, V3) for a seven-day inoculation access period at 24 ± 2 °C with a photoperiod of 14/10 L/D, respectively. The psyllids were then removed from the trees and mortality was assessed. Thereafter, psyllids and plants (six and 12 months after inoculation) were tested for *C*Las detection by qPCR. Forty to 42 sweet orange nursery trees were used per treatment.

The formula given by Gibbs and Glower [43] was used to calculate the probability of *C*Las transmission by a single psyllid when groups of five psyllids were used to determine transmission efficiency.

### 2.5. Detection of CLas by qPCR

#### 2.5.1. DNA Extraction

Total DNA was extracted from the sweet orange nursery trees and *D. citri* using the CTAB method (cetyltrimethyl ammonium bromide buffer) of Murray and Thompson [44]. The DNA from *D. citri* samples was extracted from individual adults (EPG experiment, Section 2.3) and the batches of five adult insects (transmission experiment, Section 2.4), whereas the plant DNA was extracted from 0.5 g of plant tissues from each plant (leaf midribs and petioles) in both experiments. In each round of plant and psyllid DNA extraction, negative samples were taken (*C*Las-free plants and insects) as controls. The plant and psyllid DNAs were eluted in 50 and 30 µL, respectively, of Milli-Q filtered water and stored at −20 °C until further use. The concentration and purity of the extracted DNA were measured spectrophotometrically using a Nanodrop 1000 spectrophotometer (Thermo Scientific, Wilmington, DE, USA). 

#### 2.5.2. qPCR Analysis

A total of 340 insects and 676 plant samples were analyzed by the TaqMan qPCR assay to determine the presence of *C*Las. Each qPCR assay included negative and positive controls for the target and internal control sequences, according to the method described by Li et al. [7], with slight modifications. The qPCR reaction was processed in a total volume of 12 µL, containing the master mix, 1 µL of plant tissue DNA (100 ng/µL) or 3 µL of insect DNA, 500 nM of each primer (HLBas and HLBr), and 200 nM of probe (HLBp) targeting the 16S rDNA of *C*Las. The amplification protocol consisted of an initial step at 52 °C for 2 min and 95 °C for 10 min, followed by 40 cycles of denaturation at 95 °C for 15 s and annealing and extension at 58 °C for 60s [7] in a StepOnePlus thermocycler (Applied Biosystems). The quantification Cycle threshold (Ct) values were adjusted using the StepOnePlus software and reactions were considered positive for the target sequence if the Ct value was ≤35.0; the values above 35.0 were considered negative.

### 2.6. Statistical Analyses

All the behavioral variables obtained by the EPG recording and transmission assay were transformed before analysis by sqrt (x + 1) and checked for normality using the Shapiro–Wilk W test. The non-parametric Kruskal–Wallis H test was used as most variables did not follow a normal distribution. Pairwise comparisons between the treatments were made by the non-parametric Student–Newman–Keuls test (*p* < 0.05); pairwise comparisons between the proportions of individuals that produced a given waveform type (PPW) under different treatments were analyzed using the Chi-square (*x*^2^) test (*p* < 0.05). The transmission rates of the different treatments were compared with each other using the Chi-square test and Fischer´s exact test (*p* < 0.05) when the expected values were lower than five. The statistical analyses were conducted using the SPSS version 21.0 software package (SPSS statistical package, Chicago, IL, USA) and the BioEstat version 5.0 software package (BioEstat 2007, Belém, PA, Brazil).

## 3. Results

### 3.1. Probing Behavior of D. citri

All *C*Las-exposed *D. citri*, regardless of the treatment, produced waveform C (stylet pathway activities) (Table 1). However, the proportion of psyllids that produced phloem-related waveforms (D+E1 first contact with phloem + salivation in phloem; and E2 phloem sap ingestion) was significantly lower on the thiamethoxam + chlorantraniliprole-treated trees than that on the untreated control ones (Table 1). The proportion of psyllids with a sustained phloem ingestion phase (E2 > 10 min) was significantly lower on the thiamethoxam and thiamethoxam + chlorantraniliprole-treated trees than that on the untreated control ones (Table 1).

Concerning the number of waveform events per insect (NWEI), the mean number of waveforms D + E1 and E2 produced by *C*Las-exposed *D. citri* was significantly lower on the sweet orange nursery trees treated with thiamethoxam + chlorantraniliprole than on those treated with imidacloprid and untreated control plants (Table 2). The number of times psyllids engaged in sustained phloem ingestion phase (E2 > 10 min) was significantly higher on untreated control than on treated plants (Table 2). There was no significant difference in waveform G (xylem ingestion) among the treatments (Table 2).

When the mean waveform duration per insect (WDI) was assessed, it was observed that psyllids remained longer in non-probing (np) activities on insecticide-treated than on untreated control plants (Table 2). No differences were found among treatments for the waveform C. However, the duration of waveforms D and E1 were significantly reduced when *D. citri* was exposed to sweet orange nursery trees treated with thiamethoxam and thiamethoxam + chlorantraniliprole (Table 2). Moreover, it was observed that the mean duration of waveform E2 was significantly shorter on insecticide-treated than on untreated control plants (Table 2), with a general reduction of 95.4%. No significant differences were observed for waveform G among the treatments (Table 2). 

Regarding the mean waveform duration per event (WDE), *D. citri* remained longer in np activities on insecticide-treated than untreated control trees (Table 2). No significant differences were found for waveforms C and E1 among the treatments; however, significant differences were observed for waveform D among treatments (Table 2). The mean duration of E2 and sustained phloem ingestion (E2 > 10 min) events was significantly longer on untreated control than on imidacloprid-, thiamethoxam- and thiamethoxam + chlorantraniliprole-treated plants (Table 2). Waveform G was not statistically compared because the number of xylem sap ingestion events was low (Table 2).

Regarding the sequential variables, from the time between the start of EPG to the first phloem contact, no significant differences were observed among the treatments, imidacloprid (233.78 ± 24.07 min), thiamethoxam (243.12 ± 28.03 min), thiamethoxam + chlorantraniliprole (308.42 ± 29.68 min) and control (211.38 ± 24.06 min) (H = 6.6736, d.f. = 3, *p* = 0.0831). However, significant differences for the sequential variables were found among treatments from the time between the start of EPG to the first sustained E2 (>10 min); it was shorter in the untreated control (235.95 ± 25.14 min) than the insecticide-treated plants (imidacloprid, 353.18 ± 24.79 min; thiamethoxam. 368.79 ± 25.50 min; and thiamethoxam + chlorantraniliprole, 378.64 ± 26.68 min) (H = 20.0443, d.f.= 3, *p* = 0.0002).

### 3.2. Feeding Behavior and CLas Transmission

The qPCR results of the plants exposed to a single psyllid for eight hours (EPG) showed low transmission rates. In each of the insecticide-treatment, only a single tree was found to be infected with *C*Las after six and 12 months of inoculation, giving a total of three infected trees out of the 144-treated ones (2.08%). Therefore, no comparisons could be made among the treatments. Of the 172 psyllids, 144 (83.72%) tested positive for *C*Las.

### 3.3. Insecticide Effects on Psyllid Mortality and CLas Transmission

**Figure 1:**
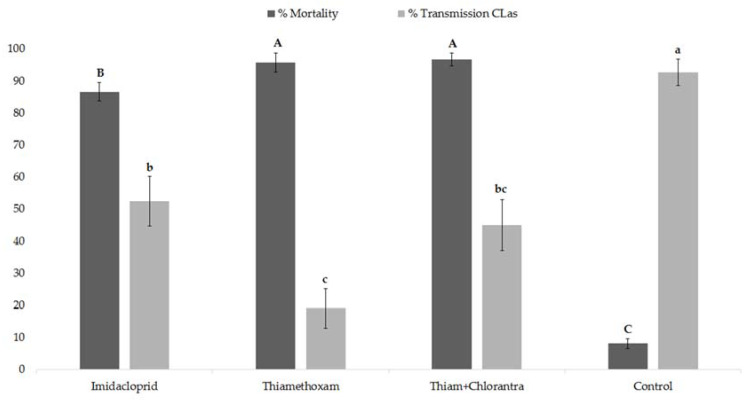
Percentage of mortality of *Diaphorina citri* and transmission rate of *Candidatus* Liberibacter asiaticus (12 months post-inoculation) on *Citrus sinensis* nursery tree treated by drench with systemic insecticides. Different upper case letters (% mortality) and lower case letters (% transmission *C*Las) represent significant differences according to the Kruskal–Wallis test followed by a Student–Newman–Keuls test (*p* < 0.05). Error bars represent standard error.

Most psyllids reared in *C*Las-infected sweet orange nursery plants tested positive for *C*Las by qPCR (167/168 (99.4%) samples with five psyllids in each). Significant differences were observed between insecticide-treated and untreated control plants after seven days on psyllid mortality (H = 123.5990, d.f. = 3, *p* < 0.0001) (Figure 1); higher mortalities were observed in plants treated with thiamethoxam and thiamethoxam + chlorantraniliprole. 

*C*Las detection after six months of inoculation resulted positive in 18 out of the 42 (42.86%) imidacloprid-treated plants, five out of the 42 (11.90%) thiamethoxam-treated plants, 13 out of the 40 (32.50%) thiamethoxam + chlorantraniliprole-treated plants, and 34 out of the 42 (80.95%) control plants. *C*Las detection after 12 months of inoculation resulted positive in 22 out of the 42 (52.38%) imidacloprid-treated plants, eight out of the 42 (19.05%) thiamethoxam-treated plants, 18 out of the 40 (45%) thiamethoxam + chlorantraniliprole-treated plants and 38 out of the 41 (92.68%) control plants (one control plant died). Thus, the systemic insecticides significantly reduced the transmission of *C*Las compared with the untreated control plants (Figure 1). Most plants that were found positive for *C*Las by qPCR showed mild leaf mottle symptoms post 12 months of inoculation (data not shown).

The estimated transmission rate (p) of *C*Las by an individual psyllid (of a group of five psyllids) on imidacloprid-, thiamethoxam-, and thiamethoxam + chlorantraniliprole-treated plants and untreated control was 13.65, 4.13, 11.27, and 41.25%, respectively.

## 4. Discussion

Vascular-restricted plant pathogenic bacteria depend on insect vectors to spread to new areas, and the epidemiology of vector-transmitted plant pathogens is strongly related to the feeding behavior of the vectors. The bacteria associated with HLB are restricted to the phloem [1], and psyllids use this vascular system as the primary source of sugar and amino acids [45,46]. The application of systemic insecticides by drench is one of the most important measures used to control *D. citri* in young groves [18]. It has been reported that systemic insecticides can reduce the transmission of plant pathogens by insect vectors in several crops [37,47,48]. The main challenge in managing HLB is to avoid and/or reduce the primary dissemination caused by immigrant *C*Las-infected *D. citri* from unmanaged areas around the commercial citrus orchard [49]. The results from the present study showed that the systemic insecticides disrupted the probing behavior of *C*Las-exposed *D. citri*, in a way that affected the *C*Las transmission efficiency, particularly by negatively affecting the activities related to the phloem phase.

The proportion of *C*Las-exposed *D. citri* individuals that reached phloem in plants treated with thiamethoxam + chlorantraniliprole was significantly lower than that of the untreated control, whereas the same in plants treated with imidacloropid and thiamethoxam alone were similar to that of the untreated control. Likewise, Miranda et al. [39] have not observed any difference in the percentages of *C*Las-free psyllids that produced waveforms related to phloem in nursery citrus trees treated with thiamethoxam and imidacloprid. On the contrary, all insecticides reduced (57–73%) the proportion of psyllids that produced sustainable phloem ingestion (E2 > 10 min), with significant differences observed on plants treated with thiamethoxam and thiamethoxam + chlorantraniliprole. No significant differences were observed in the time taken by psyllids to reach the phloem between the treated and untreated control. However, all insecticides significantly increased the time that *C*Las-exposed *D. citri* took to perform sustainable phloem ingestion.

Studies of *Ca*. Liberibacter spp. transmission by psyllids, using the EPG technique, have indicated that the inoculation phase is related to the E1 waveform [35,50]. A higher frequency and longer duration of waveform E1 correlated with a higher percentage of *C*Las inoculation by *D. citri* [50]. In the present work, the treatment with thiamethoxam + chlorantraniliprole resulted in significant reductions in the number (50%) and duration (54%) of waveform E1 per insect, whereas thiamethoxam alone had an intermediate effect (reductions of 29% and 50% in the number and duration, respectively) and imidacloprid showed a small reduction (14%) in only the duration of waveform E1. Contrary to the findings of this study, Serikawa et al. [38] had observed a significant reduction in the number and duration of waveform E1 per insect (*D. citri*) with imidacloprid treatment. However, in their study, small citrus plants (seedlings of 20–30 cm height) were used and were given imidacloprid treatment at the rate of 0.32 g per plant; the insecticide concentration was probably too high for such small plants, which may have led to the different probing behavior activities of *D. citri*.

In our study, the most pronounced effect of systemic insecticides on *C*las-exposed *D. citri* probing behavior was observed in the duration of waveform E2. In both EPG variables—duration of E2 per insect and per event—all the insecticides were able to significantly reduce phloem ingestion (≥93%) compared to the untreated control. In addition, most psyllids after ingesting phloem sap from treated plants withdrew their stylets and remained in np for long periods. Therefore, thiamethoxam + chlorantraniliprole, thiamethoxam, and imidacloprid have a deterrent effect on *C*Las-exposed *D. citri*. Similar results were obtained for *C*Las-free *D. citri* and potato psyllid *B. cockerelli*, wherein they showed a reduction in phloem sap ingestion and an increase in non-probing durations on plants treated with systemic insecticides [30,39]. 

Collectively, our EPG results indicated that all the insecticides tested, mainly the combination of thiamethoxam and chlorantraniliprole, presented a potential to reduce *C*Las inoculation by *D. citri* and therefore reduce primary spread of HLB. In the sweet orange nursery trees used in the EPG experiment, *C*L*as* was detected by qPCR only in three plants; curiously, in one plant of each insecticide treatment. This low transmission rate (2%) should be expected because of the short inoculation assess period (8 h) and the use of only one insect per plant. By using the EPG technique, Wu et al. [50] reported a transmission rate of 23% by single *C*Las-infected *D. citri*; however, in their study psyllids remained on the test plants for a 24-h inoculation access period (IAP). It has been reported that a higher number of psyllids and longer durations of IAP can improve the efficiency of *C*Las inoculation by *D. citri* [12,15]. This was also observed in our experiment with high pressure of inoculum (five psyllids per plant and a seven-day IAP), where the percentage of *C*Las-positive plants on untreated control was 93%. In contrast, insecticide-treated plants resulted in 38.8% of *C*Las-positive plants, with an overall inoculation reduction of 59%. A common characteristic observed for all the insecticides tested in this study was the significant reduction in the duration of phloem sap ingestion of *D. citri*. Although in previous studies the duration of phloem sap ingestion has not been associated with *Ca*. Liberibacter spp. inoculation [35,50], our results showed that the strong reduction in phloem sap ingestion could be also associated to a reduction of *C*Las inoculation. It is well known that during the E2 phloem ingestion phase there is also concurrent salivation that generally is ingested together with the plant sap. However, salivation during E2 is used by aphids, and possibly by psyllids, to prevent clogging of P-proteins in the food canal [51]. Such salivary secretions during the E2 phase could increase the chances of inoculation of CLas to orange trees.

Among all the treatments, thiamethoxam treatment caused the highest reduction (80%) in *C*Las inoculation by *D. citri*, followed by thiamethoxam + chlorantraniliprole (52%) and imidacloprid (44%). Butler et al. [37] reported a significant decrease in *Ca*. L. psyllaurous transmission (64%) by *B. cockerelli* with the application of imidacloprid to the soil; Ammar et al. [52] reported a reduction in the inoculation of *C*Las (80%) by *D. citri* with the foliar application of the diamide cyantraniliprole. In the present study, we had expected that the combination of thiamethoxam and chlorantraniliprole would give the highest reduction instead of thiamethoxam. One possible explanation for this could be because of confining five psyllids per plant, the advantages observed in the EPG experiment (lower proportion of individuals that reached phloem, and shorter duration and fewer events of waveform E1) were nullified. On the contrary, in well HLB-managed citrus groves, the number of psyllids per plant is very low (<0.1 psyllid per plant) [53], because all psyllids observed in these areas are from unmanaged areas (immigrating insects), the potential of thiamethoxam + chlorantraniliprole in reducing *C*Las inoculation could be higher, as observed in our EPG results.

The results of the present study demonstrated, for the first time, that the benefits of drench applications of systemic insecticides (thiamethoxam + chlorantraniliprole, thiamethoxam and imidacloprid) on sweet orange nursery trees are beyond just controlling psyllid populations. Before causing mortality, these insecticides can potentially disrupt the vector-feeding behavior, thereby, reducing the inoculation of HLB-associated bacterium *Ca*. L. asiaticus. As the experiments were performed under highly favorable conditions for *C*Las inoculation in citrus plants by *D. citri*—contrary to the field conditions, which are mostly less favorable for *C*Las inoculation—the reduction provided by the systemic insecticides could be higher in the fields than that observed in the laboratory. However, further studies are necessary to confirm this hypothesis. In general, our results provide new practical information for citrus growers that reinforces the importance of systemic insecticides in the HLB management by mainly showing the potential of these insecticides in reducing the primary dissemination in non-bearing trees.

## 5. Conclusions

Collectively, our EPG results and transmission experiments with high inoculum pressure showed that all the systemic insecticides tested (thiamethoxam + chlorantraniliprole, thiamethoxam, and imidacloprid) applied to sweet orange nursery trees are able to control *D. citri* populations and can significantly reduce *C*Las inoculation rate, therefore reducing the primary spread of HLB.

## Figures and Tables

**Table 1:** Percentage of *Diaphorina citri* that produced a specific waveform type (PPW) on *Citrus sinensis* nursery trees treated by drench with systemics insecticides, during 8-h time period.

Waveform *	Imidacloprid	Thiamethoxam	Thiam+Chlorantra	Control	χ^2^	df	*p*
C	100 (44/44) a	100 (43/43) a	100 (40/40) a	100 (45/45) a	-	-	-
D + E1	81.8 (36/44) a	67.4 (29/43) ab	47.5 (19/40) b	82.2 (37/45) a	16.002	3	0.0011
E2 E2 >10 min	77.3 (34/44) a 43.2 (19/44) ab	65.1 (28/43) ab 32.6 (14/43) b	47.5 (19/40) b 27.5 (11/40) b	80 (36/45) a 75.6 (34/45) a	12.611 8.564	3 3	0.0056 0.0357

* Waveforms: (C) salivary sheath secretion and other stylet pathway activities, (D+E1) first contact with phloem+salivation in phloem sieve tubes, (E2) phloem sap ingestion. a,b: Percentage followed by the same letter, in the same row, do not differ significantly (*p* > 0.05) using chi-square (χ ^2^) test.

**Table 2:** Means (± standard error) of EPG variables for 8 hours monitoring of *Diaphorina citri* on *Citrus sinensis* nursery tree treated by drench with systemic insecticides (20 days after application).

EPG Variables	Imidacloprid	Thiamethoxam	Thiam + Chlorantra	Control	*H*	d.f	*p*
**NPI ^a,b^**	7.48 ± 0.91 a	6.09 ± 0.65 a	5.58 ± 0.72 a	6.07 ± 0.82 a	3.6759	3	0.2987
**NWEI ^a,b^**							
np	8.32 ± 0.91 a	6.98 ± 0.65 a	6.45 ± 0.72 a	6.29 ± 0.84 a	6.8362	3	0.0773
C	8.45 ± 0.93 a	6.81 ± 0.65 a	6.18 ± 0.70 a	6.47 ± 0.86 a	6.5814	3	0.0865
D + E1	1.02 ± 0.11 a	0.74 ± 0.09 ab	0.53 ± 0.09 b	1.04 ± 0.11 a	16.5233	3	**0.0009**
E2	0.90 ± 0.09 a	0.67 ± 0.08 ab	0.50 ± 0.09 b	0.98 ± 0.10 a	14.8963	3	**0.** **0019**
NE2 >10 min	0.48 ± 0.09 b	0.33 ± 0.07 b	0.28 ± 0.07 b	0.91 ± 0.09 a	23.6247	3	**<0.0001**
G	0.02 ± NA a	0 a	0.125 ± 0.09 a	0.07 ± 0.05 a	0.201	3	0.9774
**WDI ^a,b,^** **^c^**							
np	369.05 ± 11.32 a	373.38 ± 15.82 a	386.12 ± 15.08 a	158.05 ± 21.48 b	52.3094	3	**<** **0.0001**
C	95.31 ± 11.00 a	94.26 ± 15.60 a	85.53 ± 15.30 a	95.03 ± 14.73 a	3.366	3	0.3386
D	0.68 ± 0.10 a	0.40 ± 0.07 b	0.27 ± 0.05 b	0.60 ± 0.08 a	18.0769	3	**0.0004**
E1	1.74 ± 0.27 a	1.02 ± 0.22 b	0.94 ± 0.22 b	2.01 ± 0.29 a	15.0493	3	**0.0018**
E2	13.13 ± 2.19 b	10.93 ± 2.29 bc	6.34 ± 1.42 c	219.35 ± 23.87 a	43.167	3	**<0.0001**
G	0.08 ± 0.08 a	0 a	0.80 ± 0.72 a	4.95 ± 4.50 a	0.1988	3	0.9778
**WDE ^a,b,c^**							
np	44.20 ± 4.26 a	53.52 ± 5.86 a	59.86 ± 6.47 a	25.13 ± 2.59 b	14.109	3	**0.003**
C	11.27 ± 1.07 a	13.83 ± 2.15 a	13.85 ± 2.49 a	14.69 ± 1.81 a	4.118	3	0.249
D	0.67 ± 0.05 a	0.54 ± 0.06 b	0.52 ± 0.05 ab	0.58 ± 0.06 b	8.0433	3	**0.0451**
E1	1.71 ± 0.24 a	1.38 ± 0.26 a	1.79 ± 0.32 a	1.97 ± 0.21 a	4.9619	3	0.1746
E2	14.81 ± 2.11 b	16.21 ± 2.90 b	12.68 ± 1.98 b	224.33 ± 20.88 a	65.9484	3	**<0.0001**
E2 > 10 min	21.98 ± 3.15 b	27.32 ± 4.30 b	18.19 ± 2.54 b	240.41 ± 20.20 a	61.2412	3	**<0.0001**
G	3.36 ± NA	-	6.39 ± 3.20	74.31 ± 63.95	-	-	-

a NPI—number of probes per insect; NWEI—Number of waveform events per insect; WDI—Waveform duration per insect; WDE—waveform duration per event. b Averages followed by the same letter, in the same row, do not differ significantly (*p* > 0.05) using Kruskal–Wallis test followed by a Student–Newman–Keuls test. c Values in minutes.
